# From genes to environment in shaping of an embryo: understanding embryonic-extraembryonic interactions at the BSDB autumn meeting in Oxford

**DOI:** 10.1007/s00427-019-00628-6

**Published:** 2019-02-23

**Authors:** Anna Ajduk, Elizabeth J. Duncan

**Affiliations:** 1 Department of Embryology, Faculty of Biology, University of Warsaw, Miecznikowa 1, 02-096 Warsaw, Poland; 2 School of Biology, Faculty of Biological Sciences, University of Leeds, Leeds, LS2 9JT UK

## Abstract

The British Society for Developmental Biology Autumn Meeting, held in Oxford in September 2018, was the third in a series of international workshops which have been focussed on development at the extraembryonic-embryonic interface. This workshop, entitled “Embryonic-Extraembryonic Interactions: from Genetics to Environment” built on the two previous workshops held in 2011 (Leuven, Belgium) and 2015 (Göttingen, Germany). This workshop brought together researchers utilising a diverse range of organisms (including both vertebrate and invertebrate species) and a range of experimental approaches to answer core questions in developmental biology. This meeting report highlights some of the major themes emerging from the workshop including an evolutionary perspective as well as recent advances that have been made through the adoption of emerging techniques and technologies.

It was a warm late summer day in Oxford, when 92 developmental biologists descended for the British Society for Developmental Biology Autumn meeting (September 10th–13th) held at Corpus Christi College at the University of Oxford. Organisers (Kat Hadjantonakis (Sloan Kettering Institute, US), Kristen Panfilio (University of Warwick, UK; University of Cologne, Germany), Tristan Rodriguez (Imperial College London, UK), Susana M.Chuva de Sousa Lopes (Leiden University Medical Centre, Netherlands) and Shankar Srinivas (University of Oxford, UK)) had put together a diverse and dynamic meeting programme. This meeting built on the success of the two previous meetings (Downs 2011; Stern 2015) and included an expanded contribution from researchers using invertebrate and non-model vertebrate systems. Indeed, participants presented not only the state of art knowledge on the interplay between embryonic and extraembryonic tissues, but also bridged diverse invertebrate and vertebrate animal models and illustrated the utility of advanced cell and molecular biology approaches for addressing core questions in developmental biology.

## Embryonic or extraembryonic? Cell fate specification in the early embryo

One of the core scientific questions recurring during the meeting was how cells are directed towards embryonic or extraembryonic fate. In case of mammals, the initial decisions are made in preimplantation embryos, where blastomeres first “decide” whether to form an inner cell mass (ICM) that gives rise to the future embryo body and extraembryonic membranes, or a trophectoderm (TE) that will create the embryonic part of placenta. Later, differentiation events occur inside the ICM itself, with specification of EPI and primitive endoderm (PE) lineages. EPI gives rise to the embryo proper together with extraembryonic membranes, including the allantois and amnion, and PE gives rise to the endodermal layer of the yolk sac. Many talks were also dedicated to other key developmental events, such as anterior-posterior (A-P) or dorsal-ventral (D-V) axis formation, gastrulation, or neurulation, both in mammals and non-mammalian species.

The meeting was opened by Elizabeth Robertson (University of Oxford, UK), who discussed some of her seminal work on Nodal signalling in the segregation of embryonic and extraembryonic tissues in early post-implantation development of mouse embryos, including her exciting recent studies demonstrating that Smad2/3 are required to maintain distinct embryonic and extra-embryonic cell identity in the EPI, during lineage priming (Senft et al. 2018). Elizabeth’s talk also reinforced the insight that can be gained from using integrating different approaches, such as ATAC-seq (Nelson et al. 2017), embryonic stem cells, and knockouts (Senft et al. 2018), to understand fundamental biological questions; this set the scene for what was an on-going theme throughout the meeting.

Several talks addressed the earliest stages of embryo linage specification; in particular, Miguel Manzanares (Centro National de Investigaciones Cardiovasculares, Spain) presented his results on role of Notch signalling in ICM vs. TE differentiation in mouse (Menchero et al. 2018). Takashi Hiiragi (European Molecular Biology Laboratory, Germany), on the other hand, used mouse blastocysts displaying pulsatile shape changes as an experimental model to investigate the interplay between embryo size, TE biomechanical properties and TE functionality (Chan et al. 2018). Véronique Azuara’s (Imperial College London, UK) talk revealed the role of the BMI1 transcription factor in EPI/PE specification in mouse blastocysts. Claire Chazaud (GRed Research Centre, France) showed how experimental work can be complemented by mathematical modelling in order to dissect a role of Nanog, Gata6 and FGF signalling in differentiation of murine ICM cells (Tosenberger et al. 2017). Nestor Saiz from Kat Hadjantonakis’ group (Sloan Kettering Institute, US) discussed a potential mechanism that links EPI/PE lineage size with fate decisions of individual ICM cells. Ayaka Yanagida from Jennifer Nichols’ and Kevin Chalut’s groups (University of Cambridge, UK) focused on the relationship between biomechanical properties of the EPI/PE progenitor cells and their ability to segregate into the separate EPI and PE layers, typical for a mature mouse blastocyst. Nicolas Porchet from Jérôme Collignon’s lab (Institut Jacques Monod, France) described involvement of Nodal signalling in PE specification in mouse embryos.

Progressing to the post-implantation stages, Matthew Stower from Shankar Srinivas’ group (University of Oxford, UK) and Go Shioi from Yasuhide Furuta’s lab (RIKEN, Japan) showed how fluorescently tagged proteins and time-lapse imaging helped in revealing the role of cellular rearrangements in the formation of anterior visceral endoderm (AVE) and distal visceral endoderm (DVE) in mouse embryos (Shioi et al. 2017). Di Hu, again from Srinivas’ group stayed in the mouse theme and discussed the role of Ets2 transcription factor in extraembryonic ectoderm (ExE) and AVE specification. Jennifer Nichols (University of Cambridge, UK) gave a comprehensive talk about functions of Oct4 in mammalian embryonic development, starting from preimplantation stages through A-P axis formation to gastrulation (Mulas et al. 2018). Gastrulation, and particularly involvement of Nodal signalling in this process, was also the main topic of Vasso Episkopou’s (Imperial College London, UK) talk, presenting the dose-dependent functions of Nodal (Carthy et al. 2018). Following on from this, Elisabetta Ferretti (Novo Nordisk Center for Stem Cell Biology, Denmark) discussed the molecular mechanisms of mesoderm formation.

The 2018 Dennis Summerbell Award Lecture was delivered by Mariya Dobreva (VIB-KU Leuven Centre for Brain and Disease Research, Belgium). This award was given for Mariya’s detailed and elegant lineage tracing experiment that defined the developmental origins of the amnion, the innermost embryonic membrane. Mariya’s analyses indicate that the amniotic ectoderm arises from four types of progenitor cells residing in the early proximal anterolateral epiblast and that Smad5 has an inductive role in mediating spatial cues crucial for establishment of the amniotic ectoderm (Dobreva et al. 2018).

Although the mouse is still the predominant model for scientists interested in preimplantation embryonic development, other mammalian species made an appearance at this meeting as well. A significant part of the conference was dedicated to marsupial, rabbit, bovine and even human preimplantation embryos. A highlight was Stephen Frankenberg’s (University of Melbourne, Australia) talk on his pioneering research regarding embryonic lineage differentiation in marsupials (wallabies and dunnarts), focusing mostly on a potential role of Gata2 in TE formation. James Turner (Francis Crick Institute, UK) talked about his ground-breaking research using single-cell RNA-seq in marsupial (opossum) embryos to search for linage specification markers and mechanisms of X-chromosome dosage compensation. Berenika Plusa (The University of Manchester, UK) compared roles of Nanog, Gata6 and FGF signalling in mouse, rabbit and human embryos and demonstrated differences in lineage specification mechanisms between these species. The rabbit story was continued by Anna Piliszek (Institute of Genetics and Animal Breeding, Polish Academy of Sciences, Poland), whose results reveal that ICM cells in rabbits differentiate later than in mouse embryos (Piliszek et al. 2017). Zofia Madeja (Poznan University of Life Sciences, Poland) discussed the relationship between chromatin territory structure and gene expression (Orsztynowicz et al. 2017), and also the signalling pathways involved in maintaining pluripotency in bovine epiblast cells and embryonic stem cells (ESCs) (Madeja et al. 2015). Moving further through embryogenesis, Monika Bialecka from Susana M. Chuva de Sousa Lopes’ lab (Leiden University Medical Center, the Netherlands) talked about PGCs differentiation in in vitro cultured human embryo outgrowths. Siegfried Roth (University of Cologne, Germany) presented diverse modes of D-V axis specification in various groups of insects, focusing on the intricate interplay between Toll and BMP signalling (Sachs et al. 2015). Federica Bertocchini (Instituto de Biotecnologia de Cantabria, Spain) talked about A-P axis formation in chicks (Arias et al. 2017) and its evolutionary analogies in reptiles, whereas Irene Yan (Universidade de São Paulo, Brazil) discussed transcriptional and post-transcriptional regulation of Scratch2, a conserved regulator of neural development, in chick neurulation.

## Extraembryonic membranes at the interface: From environment to genes to phenotype

In all animals, extraembryonic membranes have important functions including facilitating gas and nutrient exchange and protecting the embryo from mechanical stress. There are, however, key differences between animal groups in how the extraembryonic membranes are formed and how they function. Myriam Hemberger (Babraham Institute, UK) talked about her project systematically identifying mutations in mice associated with placental defects (Perez-Garcia et al. 2018) and how she uses this approach to discover novel pathways of trophoblast differentiation and placentation. In mammals, placental function in particular has been associated with growth of the embryo, and environmental perturbations, including stress, are known to affect both the placenta and embryo itself. Rosalind John’s (Cardiff University, UK) pioneering research has implicated placental imprinting in embryonic growth but also mediating maternal care (Creeth et al. 2018). Additionally, as demonstrated by David Harrison working with Rosalind, placental imprinting may also alter offspring phenotypes.

The effects of environmental exposures, such as alcohol, on function of extraembryonic membranes was discussed by Jacinta Kalisch-Smith (University of Queensland, Australia) and Diana Laird (University of California, US) extended this discussion to incorporate how environmental exposures might affect subsequent generations, possibly via epigenetic mechanisms. Elizabeth Duncan (University of Leeds, UK) discussed the role of epigenetic mechanisms in how insects, like the honeybee, are able to respond to environmental cues to produce two or more entirely different phenotypes from the same genotype, a phenomenon known as phenotypic plasticity.

In insects, there are generally two extraembryonic membranes, the amnion and the serosa. Kristen Panfilio (University of Cologne, Germany; University of Warwick, UK) discussed what we know about the developmental origins of these tissues in insects and analogies with mammalian extraembryonic membranes. Kristen also delved into her work on the rupture of extraembryonic membranes (Hilbrant et al. 2016), a normal part of embryogenesis in insects, but a pathological process in mammals. In addition to the key roles in morphogenesis, Maurijn van der Zee (Leiden University, the Netherlands) emphasised that the serosa protects the insect eggs against environmental exposures including pathogens and desiccation (Jacobs et al. 2014). This was reinforced by Nora Braak (Oxford Brookes University, UK), who showed that the serosa, although developing differently in butterflies, also has a role in embryonic immunity.

## Cutting edge new methods applied to solving old puzzles

The overarching theme of the talks was the increasing contribution of cutting edge technologies to further advancement of developmental biology research. A good example here are the highly sophisticated imaging techniques, often combined with transgenic reporters and advanced computational image analysis that help to visualise dynamics of developmental processes, as demonstrated by Matthew Stower and Kristen Panfilio (light-sheet microscopy), Go Shioi (spinning-disc microscopy) and Anna Ajduk from University of Warsaw, Poland (optical coherence microscopy) (Karnowski et al. 2017).

Another cutting-edge experimental approach that has enormous future potential is single-cell RNA sequencing, allowing researchers to pinpoint transcriptional changes occurring in individual cells at multiple time points in the development. Its utility for different organisms and biological questions was clearly illustrated in talks by James Turner, Di Hu, Elisabetta Ferretti and also by Sarah Teichmann (Wellcome Sanger Institute, UK). Sarah’s pioneering work using singl-cell sequencing is helping to reveal how cell states and cellular phenotypes change during normal and pathological processes in various biological contexts, including in the immune system (Hagai et al. 2018) and in the maternal-fetal interface (Vento-Tormo et al. 2018) and is contributing to the Human Cell Atlas project (https://www.humancellatlas.org/).

Staying with the molecular biology theme, ‘omics research methods, for example ATAC-seq to identify putative enhancer regions and ChIP-seq to identify binding sites of transcription factors, are making massive contributions to our understanding of gene regulation during development, and this was clearly demonstrated by several talks including our plenary speaker Elizabeth Robertson and also Laura Banaszynski (UT Southwestern Medical Center, US).

It was also clear that recent and unprecedented development of effective ways to produce transgenic animals resulted in a number of projects dedicated to a widespread functional analysis of mammalian genes. Myriam Hemberger and Jaime Rivera-Perez (University of Massachusetts Medical School, US) reported on the projects looking for various embryonic lethal mutations in mice, focusing on placental (Perez-Garcia et al. 2018) or embryonic phenotypes, respectively.

Another approach that is increasingly popular in developmental biology and definitely complements a more traditional way of experimental embryo work is an in vitro culture system for peri- and post-implantation embryos or for differentiating ESCs (Jennifer Nichols, Monika Bialecka, Elisabetta Ferretti). Indeed, Ali Brivanou’s (Sloan Kettering Institute, US) closing keynote lecture was not only a great summary of cell fate specification mechanisms, but also a very interesting introduction to in vitro models of gastrulation and neurulation. He presented results on the role of BMP4/Wnt/Nodal pathway and TGFβ signalling, respectively, in these processes (Yoney et al. 2018). He also discussed the potential of creating a synthetic 3D embryo built exclusively from cells originating from ESCs and the contribution that this model would have to our understanding of developmental biology (Metzger et al. 2018).

## An evolutionary perspective and future prospects

By gathering researchers representing various branches of developmental biology, working on different animal models and stages of embryogenesis, the meeting organisers created a unique platform for broader scientific discussions, including a challenging, but yet crucial for developing a deeper understanding of developmental processes, evolutionary perspective. Kristen Panfilio set the scene for evolutionary comparisons in her talk early in the meeting, discussing the analogies between insect and mammalian extraembryonic membranes. Although these tissues have different evolutionary and developmental origins, they can be considered functionally analogous. Indeed, it has been proposed that the evolution of extraembryonic membranes in insects facilitated the radiation of insects on land (Jacobs et al. 2013; Zeh et al. 1989) as one of these membranes, the serosa, protects against desiccation (Jacobs et al. 2013). Similarly, the evolution of the amniote egg has been implicated in supporting the radiation of vertebrates on land (reviewed in Ferner and Mess 2011).

The field of evolution and development (Evo-Devo) has highlighted aspects of development that are conserved amongst phylogenetically diverse animals and therefore were likely present in the bilaterian ancestor; for example the role of BMP signalling in dorsoventral axis specification and the subdivision of the dorsoventral axis into distinct ectodermal domains (reviewed in Bier and De Robertis 2015). However, questions remain over how novelties, such as the extraembryonic membranes of insects and vertebrates, evolved and the molecular mechanisms that underpin these novelties. Understanding the molecular and evolutionary origins of the extraembryonic membranes and the intricate interplay between these membranes and the embryo in normal development and morphogenesis, in a phylogenetically diverse range animals, will allow us to examine the extent to which these molecular mechanisms overlap or converge. These comparisons may allow general inferences to be made about the evolution of novel cell types, cell fate and lineage specification as well as the role of co-option of conserved cell signalling pathways such as Wnt, FGF and Notch (Pires-daSilva and Sommer 2003) and gene regulatory network, transcription factor, genome organisation and chromatin landscape evolution in these processes.

In amniotes, we understand most about early development in mice, and in insects, we know most about development in the fruit fly, *Drosophila melanogaster* although our knowledge of other species is increasing. We now have access to an array of cutting-edge techniques that can be readily applied to a wide range of organisms, including CRISPR/Cas9-mediated genome editing, live imaging technologies and advances in ‘omics technologies like single-cell sequencing. Applying these tools will rapidly advance this research field and deepen our understanding of early development, including the specification and function of the extra-embryonic membranes, the intricate interactions between the embryo and these membranes in normal and pathological states, how robust or sensitive these developmental processes are to environmental stressors, as well as how these remarkable systems evolved independently in vertebrates and insects.
